# Interfacial electrofluidics in confined systems

**DOI:** 10.1038/srep26593

**Published:** 2016-05-25

**Authors:** Biao Tang, Jan Groenewold, Min Zhou, Robert A. Hayes, Guofu (G.F.) Zhou

**Affiliations:** 1 Electronic Paper Display Institute, South China Normal University, Higher Education Mega Center, Guangzhou 510006, P. R. China; 2 Van ‘t Hoff Laboratory for Physical and Colloid Chemistry, Debye Research Institute, Utrecht University, Padualaan 8, 3584 CH Utrecht, The Netherlands

## Abstract

Electrofluidics is a versatile principle that can be used for high speed actuation of liquid interfaces. In most of the applications, the fundamental mechanism of electro-capillary instability plays a crucial role, yet it’s potential richness in confined fluidic layers has not been well addressed. Electrofluidic displays which are comprised of thin pixelated colored films in a range of architectures are excellent systems for studying such phenomena. In this study we show theoretically and experimentally that confinement leads to the generation of a cascade of voltage dependent modes as a result of the electro-capillary instability. In the course of reconciling theory with our experimental data we have observed a number of previously unreported phenomena such as a significant induction time (several milliseconds) prior to film rupture as well as a rupture location not corresponding to the minimum electric field strength in the case of the standard convex water/oil interface used in working devices. These findings are broadly applicable to a wide range of switchable electrofluidic applications and devices having confined liquid films.

Electrofluidics, also known as electrowetting, has its origins in electrocapillarity dating back to 1875^1^. Only recently, however, have a large range of applications and devices including patterning^2^, lab-on-chip^3^, liquid lenses^4^,^5^ and displays^6^ been proposed and developed based on the principle. The availability of suitable dielectric materials and coatings has been a key enabler in this regard^7^,^8^. Amorphous fluoropolymers provide the required fluidic reversibility for these capacitive devices^9^. Apart from technological interest wetting dynamics is still an active field also from a fundamental point of view^10^,^11^.

The device architecture described in this paper consists of a vast array of repeating electrofluidic cells, which in the case of an electrofluidic display serve as pixels^6^. The pixel wall structure is used as a means of controlling the (colored) oil location during both liquid dosing as well as device operation (Fig. 1). The behavior of the oil/water interface and its interaction with both the fluoropolymer and wall materials is critical to the performance of the electrofluidic device (Supplementary Information section 1). When an electric field is applied to a molten insulating polymer layer that has a dielectric mismatch with the (air) gap on top of the layer, undulations with wavelengths from 100 nm to several microns via an electro-capillary instability can be generated^12^,^13^. More classical work on electrocapillarity consists of a liquid metal layer that is countered with an electrode parallel to the liquid across a vacuum gap typically on the centimeter scale^14^,^15^. In ref. [16] a similar situation is described but for liquid metal instead of water as a conducting liquid and in place of the vacuum gap transformer oil was used. Therefore the device under study here has very strong similarities in terms of more classical work on electro-capillarity be it on different length (0.1 mm) and voltage (tens of Volts) scales.

Under application of a potential, the conducting liquid (in our case water) allows the surface charge to accumulate at the (oil-water) interface, which leads to an electrostatic compressive pressure across the gap or oil film. Provided that the potential is high enough an undulation in the oil film forms which will grow in time until the oil film ruptures. Counteracting the electrostatic pull on the oil film is a capillary restoring force^12^,^13^,^14^,^15^. In a system without confinement it has been established that the wavelength of such undulations is described by the fastest growing (unstable) mode of a spectrum of electro-capillary-waves^12^,^13^,^14^,^15^. Large wavelengths take longer to develop compared to small ones as a result of the friction in the liquid film while for shorter wavelengths the capillary restoring force impedes the effectivity of the electrostatic driving force.

In an unconstrained film one finds a continuous spectrum of waves depending on the film thickness and applied voltage^17^. For liquid metals this confinement effect has been addressed^18^,^19^ in a circular, ring and infinite strip geometry. In our work, the electro-capillary instability is studied in the context of lateral confinement of the liquid film in square and rectangular cells. As a result of meniscus pinning to the pixel wall the typical wavelengths that can develop in the instability are expected to be discretized^18^,^19^. This enables us to define transition voltages between different modes and also leads to a threshold voltage^18^,^19^,^20^ below which the film remains stable. The phenomena reported by Sun & Heikenfeld^21^ are possibly a result of the mechanism described above. Here we provide a comprehensive study of electrofluidic modes selection.

The theoretical part of the study reported here focuses on the underlying onset of instability in the oil film as opposed to late stage^9^ and comprehensive^22^ modeling attempts. This onset leads to a rupture of the oil film so water will contact the base fluoropolymer layer. Due to the electrowetting effect the film will contract further up to the point such that locally everywhere the contact angle is at the advancing value. This has been reported^6^ for electrowetting-based switchable optical elements.

We have conducted a high speed camera investigation of electrofluidic display pixels. We report here for the first time two key observations. Firstly the initial rupture of the oil film does not occur at the pixel wall, the precise location of film rupture depending on applied voltage. Secondly, we have noted an induction time of several milliseconds between the application of the voltage and the rupture of the oil film (Fig. 2). The induction time does not appear to be strongly dependent on the thickness of the precursor oil film within the limited range of thicknesses explored in this study. To provide a fuller understanding of the behavior of oil/water interfaces in such confined systems we applied the electro-capillary wave model to confined systems and reconciled it with a broad range of experimental observations.

Assume the reference state is a flat configuration of the oil film. Consider small deflections from the reference interface shape:


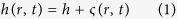


The pressure acting on the film is composed of a capillary and an electrostatic component. In our system it is observed that the oil film thickness (5 μm) is much smaller compared to the observed undulation wavelengths (>50 μm). Therefore the analysis can be considerably simplified because under the large wavelength condition the lateral components of the electric field can be neglected. In most previous studies^14^,^15^,^16^,^18^,^19^ of the electro-capillary instability the dielectric gap is comparable or large compared to the wavelength, a condition under which the lateral components of the electric field cannot be neglected. If one also assumes that the undulation is relatively small compared to the film thickness the pressure on the oil film is written as:


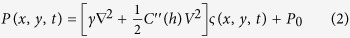


Positive values of this pressure promote film growth. *C*(*h*) is the capacitance of an oil film of thickness *h*, γ is the oil/water interfacial tension and *V* is the voltage applied to the system. The first term on the right hand side is a capillary restoring pressure while the second term is the electrostatic component of the pressure that tends to destabilize the film. The second derivative of the oil film capacitance gauges the way the electrostatic pressure is modulated as a result of infinitesimal changes in the height of the oil film^12^,^13^.

This expression (Eq. 2) for the pressure is valid in the rectangular domain within which the liquid is pinned. Outside the domain the reference pressure is zero. The pressure jump *P*_0_ that occurs in the domain is there to ensure volume conservation. This method to deal with volume conservation has been adapted from^18^,^19^.

If the pressure is known for given change of the meniscus shape the fluid flow is governed by the lubrication approximation^12^,^13^ of the Stokes equation valid for a thin liquid film


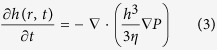


It is justified to neglect the drag by the water in the upper layer since the water layer is much thicker so the drag is expected to be significantly lower compared to the thin oil film.

In this study square and rectangular cells are considered with lateral dimensions 150 × 150/150 × 315/67.5 × 315 microns and height dimensions defined by pixel wall structure 5.5/5.7/5.6 microns respectively. The short lateral dimension will be denoted by *L*_*x*_ and the long dimension by *L*_*y*_. Natural modes in this geometry are denoted by [*m*, *n*] and are described by the function:


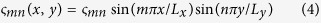


These modes satisfy the pinning boundary conditions. However the modes with both *m* and *n* uneven do not satisfy volume conservation, therefore in these cases considering pure modes is not justified. This shortcoming can be addressed by invoking the pressure jump *P*_0_, which gives rise to a small discrepancy compared to the pure mode analysis, which is discussed in Supplementary Information section 3. The thin film evolution equation can be used to derive the time evolution of an initial perturbation for each mode. The modes are characterized by a wavenumber that must be compatible with the pixel dimensions:


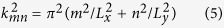


The amplitude is governed by exponential growth (or shrinkage in the case of a negative time constant) with the following rates


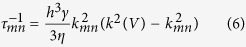


The electrostatic driving force is also written as a wavenumber:


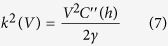


Inserting the typical values for this system in Eq. (6) using Eq. (7) gives a timescale of several milliseconds. Interestingly, the height of the oil film is hardly affecting the growth rate since the hydrodynamic friction scales with the cube of the oil film height while the driving force scales as *C*''(*h*) ~ *h*^−3^, resulting in a constant growth rate. We do find however a strong dependence of the growth rate on applied voltage. Both these observations are at least qualitatively borne out by observation.

Now, from an energetic point of view positive growth rates can be realized whenever *k*(*V*) > *k*_*mn*_. This marginally unstable energy criterion defines the voltage 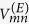. One can also consider mode selection from a dynamic perspective: which of all possible modes develops fastest. Dynamic transitions between a mode [*m*, *n*] and [*m*′, *n*′] are defined at the point where the growth rate of mode [*m*, *n*] equals that of the neighboring mode [*m*′, *n*′]. This dynamic criterion translates into: 
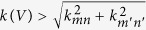
, which defines the onset of the dynamic transition at the voltage of 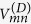.

In systems exhibiting an instability without external forces, the fastest growing mode is the one that is selected.

In the cells under study here, the wall is expected to exert an additional force on the interface close to it, as a result of fringe electric fields arising from a dielectric mismatch between the photoresist walls and the neighboring oil. In addition there may be other sources of inhomogeneity that can act as a trigger, such as over- or underfilling of a pixel or inhomogeneous pinning of the contact line by the photoresist. In case of sufficient external trigger we assume it is possible to have mode selection of marginally unstable modes.

With this in mind we anticipate that the transition from one mode to the other should be found between the dynamic transition or at the marginally unstable point. To underline the theoretical universality of the transition we express the transition voltages normalized by


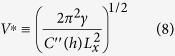


which is a characteristic voltage of the system. For the capacitance the simple formula for a parallel plate capacitor is taken of the two layer system oil and fluoropolymer. This is justified if the water is sufficiently conducting such that the interface can be an equipotential one at the value of the applied potential.

We determined the statistical distribution of occurring modes for each sample on an array of 7 × 7 cells. The modes have been identified by analyzing the location and the number of minima in the oil film thickness that evolve in each cell shortly after the voltage switching occurs (Supplementary Information section 2.2). In Fig. 3 the results summarized for the different modes are depicted together with their respective occurrence for each mode on the sample with 150 × 315 μm^2^ cells. In this case we have found the modes: [1, 2]/[1, 3]/[1, 4]/[1, 5] and [2, 5]. The 150 × 150 μm^2^ cells showed modes [1, 2]/[1, 3] and [3, 3] (Supplemental Figs 1 and 3) while the 67.5 × 315 μm^2^ cells showed modes [1, 2]/[1, 3]/[1, 5] and [1, 7] (Supplemental Figs 2 and 5). Note that the use of mode [1, 3] in the case of the 150 × 150 μm^2^ is shorthand for the more correct notation: [1, 3] + [1, 3], which is more consistent with the observed symmetry of this mode (Supplemental Fig. 6). In Fig. 4 the experimental and modeled values of the transition are shown for all samples combined in terms of the experimental and theoretical transition voltages between the modes.

The value of the interfacial tension used to compare the theoretical transition voltages with the measured ones is fixed at 22 mN/m and leads to the best correspondence with the [1, 2] mode in the 150 × 150 μm^2^ pixel. This value is within the experimental error of the measured interfacial tension. The [1, 2] mode in the 150 × 150 μm^2^ pixel is most suitable for comparison with theory since there is no discussion about whether it is marginally unstable or fast growing; if we wait long enough the dynamic transition and marginally stable transition coincide. For the [1, 2] mode we therefore expect that it is well described by the marginally stable condition. Accepting this value of the interfacial tension we find that all other transitions are also well described by the marginally stable condition. This points to a trigger via forces exerted by the wall on the liquid interface as described earlier. For 67.5 × 315 μm^2^ mode [1, 7] is observed and [2, 5] is not observed while for 150 × 315 μm^2^ mode [2, 5] is observed but not mode [1, 7]. This is entirely consistent with the theory underlying the mode selection. In the model, for pixel aspect ratio larger than around 2.8 we find that both the dynamic and the marginally unstable transition voltage of the [1, 7] mode become lower compared to the [2, 5] mode.

One observation remains puzzling. In the 150 × 315 μm^2^ pixels the exact sequence of observed modes as a function of voltage is [1, 2]′ →[1, 3]′ →[1, 2] →[1, 3] →[1, 4] →[1, 5] →[2, 5] (Supplemental Fig. 4 and Video) and for 67.5 × 315 μm^2^ it is observed to be [1, 3]′ →[1, 2] →[1, 3] →[1, 4] →[1, 5] →[1, 7] (Supplemental Fig. 5 and Video). So prior to the expected sequence some modes (indicated with a prime) are triggered not described by the linear stability analysis used here.

The summary of our measurements combined with the model in Fig. 4 show that apart from the ‘primed modes’ discussed above all modes show up in the predicted order and all but the [1, 3] mode of the square pixel are consistent with the marginally unstable criterion. The marginally unstable criterion suggests that there are external forces triggering and preventing the modes that would normally be the fastest growing. The triggering forces can have several origins. Close to the wall fringe electric fields resulting from the dielectric mismatch between the pixel walls and the oil can be expected to exert highly localized forces on the interface. Another trigger could be non-planar shapes of the interface due to inhomogeneous pinning of the contact line near the photoresist or a small amount of over- or under-filling of oil. We have not yet analysed the effect of fringe fields or interface inhomogenieties quantitatively. We speculate that the ‘primed modes’ that occur prior to the expected sequence may be the result of either one of the triggers described above. Another interesting observation is that the [1, 3] mode is apparently not responding to the marginally unstable criterion but is found at a higher voltage than predicted. It is interesting to note that the interface shape corresponding to this particular mode is such that it is depleted in oil everywhere near the wall, while in all other observed modes we find alternating regions of depletion and excess of oil along the wall (Supplemental Fig. 6). This could potentially be related to its exceptional position in terms of transition voltage, yet again without better knowledge of the wall and inhomogeneity effects, this statement is speculative which we would like to address in more detail in the future. A final point of discussion in terms of mode selection is that many modes, which are theoretically possible, are not observed in practice. Again, the absence of certain modes in practice could be resulting from triggering forces in combination with the varying degrees of growth rates for the competing modes, conspiring to suppress the unobserved modes. We plan to address these issues in the future both from a modeling perspective and experimentally. For the modeling it would be of interest to include the triggering forces and interface inhomogenieties to see how it will affect the selection of instabilities. On the experimental side one could for instance try to match the dielectric constant of the photoresist with that of the oil, such that the fringe fields can be minimized.

The open items described above should however not cloud the level of insight that has been obtained in this study. The work provides convincing empirical evidence for the existence of discretized modes in confined electro-capillary fluidic systems. Moreover we find that the data can for the greater part be explained by analytic stability analysis without adjustable parameters. The insights obtained in this work, apart from being of fundamental interest, will also enable further optimization of devices based on this mechanism including for example, grey scaling in electrofluidic displays.

## Methods

Amorphous fluoropolymer (AF1600, Chemours, ε_r_ 1.934) was used as insulator (thickness *d* ≈ 0.85 μm) material. The insulator was spin-coated on ITO/glass substrates with a resistivity of 100Ω Sq. The pixel walls were formed from an n-type photoresist (SU8-3005, Microchem) by lithography. As a result the fluoropolymer coated only the base of the pixels and not the pixel walls. The fluoropolymer surface was activated using a reactive ion etch (RIE) to facilitate the coating of the photoresist. Prior to liquid dosing the engineered substrate was heated above the Tg of the fluoropolymer (160 °C) to return it to its hydrophobic state. Oils are formulated by dissolving a non-polar dye in decane (C_10_H_22,_ ε_r_ 2.2). A colored oil of 0.21 M dye concentration was used in this study. The polar phase sealed in the device was 0.001 M NaCl aqueous solution. The liquids were dosed to the substrate by raster filling at low speed (~1 mm/s). The cells were edge-sealed using a pressure sensitive adhesive (PSA) attached to the cover plate. The interfacial tension for the colored oil/water system was experimentally determined (at 25°) to be 0.020 +/− 0.002 N/m by the drop-weight method. The oil viscosity (at 25°) was found to be 1.23 +/− 0.01 mPa.s, measured by using a rheometer (HAAKE MARS).

A high-speed camera (MIRO M110) with 1600 fps sample rate was employed for recording the electro-hydrodynamic instability phenomenon under an electric field. A series of square waves of 0.5 Hz frequency supplied by a waveform generator (Agilent 33500B Series) were applied to the oil/water system in our study.

## Additional Information

**How to cite this article**: Tang, B. *et al.* Interfacial electrofluidics in confined systems. *Sci. Rep.* **6**, 26593; doi: 10.1038/srep26593 (2016).

## Supplementary Material

Supplementary Information

Supplementary video

## Figures and Tables

**Figure 1:**
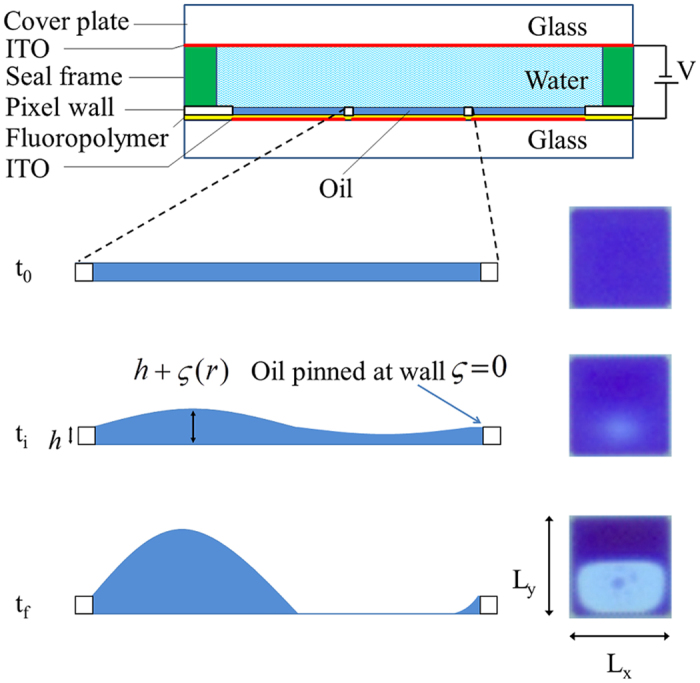
Electrofluidic cell architecture showing constituent materials and oil film profiles with corresponding top-view microscope images. The oil is contained within a wall structure that is built on an insulator covered electrode structure^6^. The non-polar oil is covered by the polar liquid, for example water, in a common fluid channel. The liquids are low in viscosity and deliver switching times on a millisecond timescale at typical cell dimensions (<0.2 mm). It is typically observed that the non-polar oil is strongly pinned at the top of the pixel wall, the radius of curvature of the wall corner being typically <~1 micron. The pixel wall material, commonly a photoresist, is crystalline in nature and chosen or prepared so that the oil does not migrate across the wall to adjacent pixels during switching since the contact angle of the water-oil interface near the photo-resist is less than 90^°^ water receding. *t*_*0*_*, t*_*i*_ *and t*_*f*_ denote the oil/water interface in a pixel off state (no applied voltage), before initiation of oil film rupture (with applied voltage), and final on state, respectively.

**Figure 2:**
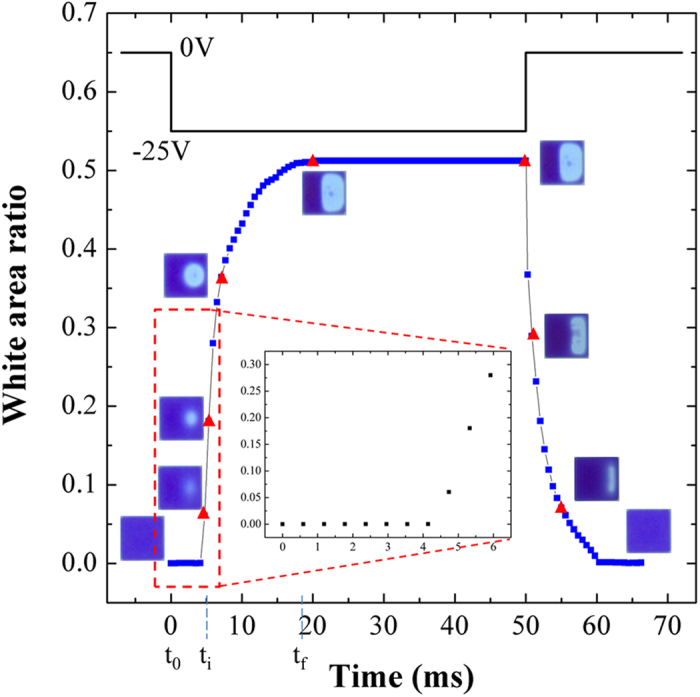
Electro-optic data as a function of time for square cells. A voltage of −25V is applied to initiate the opening of the pixels. The white area corresponds to the open pixel area. The inset shows an enlargement of the initial breakage region. Corresponding microscope images (indicated by the red triangles) of cells during film rupture and reformation are also shown. *t*_*0*_*, t*_*i*_ *and t*_*f*_ denote the oil/water interface in a pixel off state (no applied voltage), before initiation of oil film rupture (with applied voltage), and final on state, respectively.

**Figure 3:**
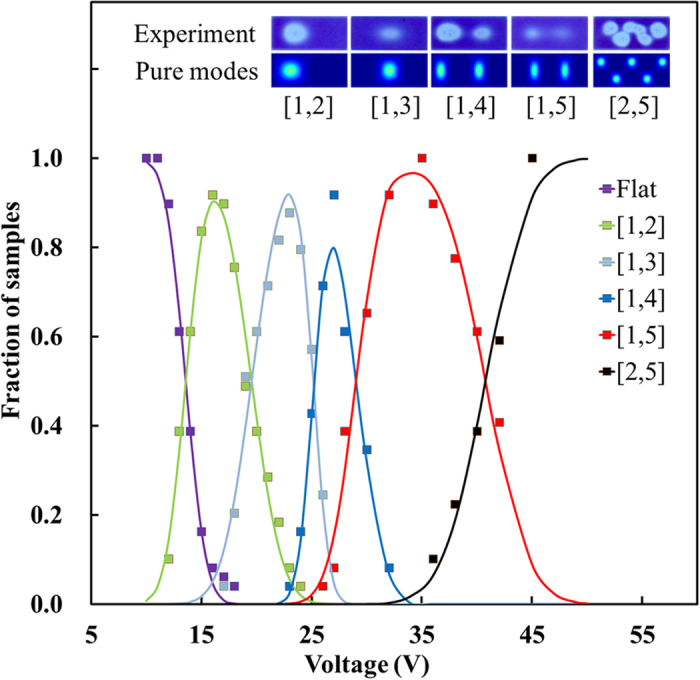
The observed count for each mode as a function of applied voltage for the 150 × 315 μm^2^ cell. Symbols represent processed experimental data and lines to corresponding data fits as described in the Supplementary Information section 2.1. Inserts at top show typical experimental images and corresponding pure modes generated by model calculation (Supplementary Information section 2.3). The experimental transition voltages and their error bars have been determined by the fit procedure.

**Figure 4:**
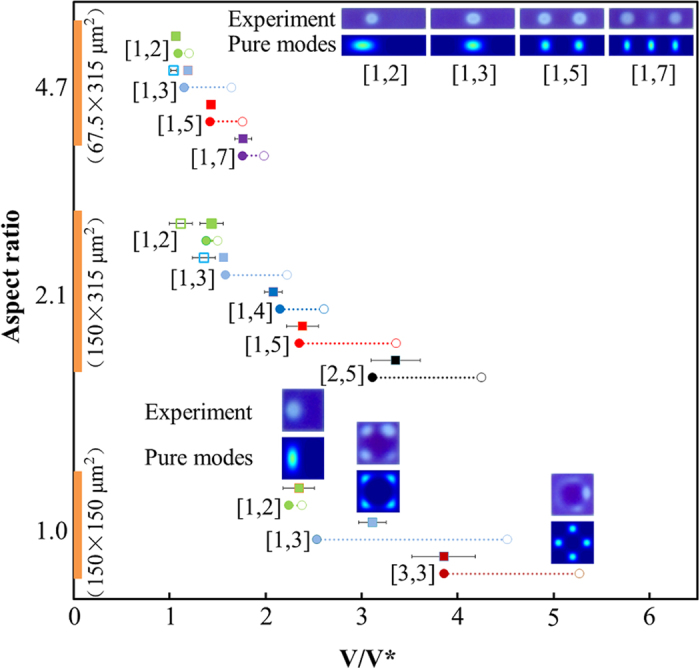
Depiction of normalized theoretical dynamic transition voltages 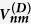 (solid circles) and marginally unstable transition voltages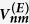 (open circles) for the different geometries and different modes within each geometry. The experimental determination of the transition voltages for each observed mode which is compatible with the theoretically expected sequence is depicted by solid squares. The unexplained primed modes [1, 2]′ and [1, 3]′ are depicted with open squares. Inserts show typical pixel microscope images of the various modes both experimental and pure. Calculated dynamic mode positions for the [1, 2] mode do not exist.
