# Supplementary material for: Interfacial electrofluidics in confined systems
Source: Sci Rep. 2016 May 25;6:26593. doi: 10.1038/srep26593 (PMC4879560; doi:10.1038/srep26593)
Supplement: Supplementary Information [file srep26593-s1.doc]

**Interfacial electrofluidics in confined systems**

Biao Tang^1^, Jan Groenewold^1,2^, Min Zhou^1^, Robert A. Hayes^1^ & Guofu (G.F.) Zhou^1^

# Supplementary information

## Background information

## Supplementary methods

1. Fit procedure for data
2. Analysis of the images
3. Eigenvalue problem for finding volume conserving modes in Matlab
4. Plotting of the theoretical unstable modes

## Supplementary discussion

Correcting for modes that are not strictly volume conserving

## 4. Supplementary video

1) Modes transition with applied voltage for square pixels (150×150 μm^2^)

2) Modes transition with applied voltage for rectangular pixels (150×315 μm^2^)

3) Modes transition with applied voltage for rectangular pixels (67.5×315 μm^2^)

## 5. Supplementary figures (Extended Data)

## Background information

## *Background information on the display aspects of the cell arrays*

## The pixel wall structure is used as a means of controlling the colored oil location during both liquid dosing as well as device operation. The oil is covered by the polar liquid, for example water, in a common fluid channel. The behavior of the oil/water interface and its interaction with both the fluoropolymer and wall materials is critical to the performance of the electrofluidic display device. The liquids are low in viscosity and deliver switching times on a millisecond timescale at typical pixel dimensions (<0.2 mm), enabling video content to be shown.

## Supplementary methods

## *Fit procedure for data*

## To determine the distribution of transition voltages for a given mode the most direct way would be to track for each cell individually where the transition occurs upon small increments in voltage. Thus a probability density distribution $p_{mn}(V)$ could be obtained. The probability to find the [m,n] mode transition to occur from its neighboring mode below it in the interval $[V,V+\Delta V]$ is then given by:

$\int_{V}^{V+\Delta V} p_{mn}(V')dV'$ (1)

Evaluating the precise location of voltage transition for a large number of cells requires small increments in voltage. In this paper we use a more convenient method to obtain the probability density function. For a number of discrete voltages a histogram is determined of how often a certain mode is detected [see figure 1 and 2 in extended data and figure 4 in the main paper]. From the data it can already be seen that upon occasion some modes do overlap. To transfer these histograms into the abovementioned probability distribution one can compare the probability function that a mode [m,n] has not been crossed by, in other words the probability that we are below the [m,n] transition:

$P_{mn}^{<}(V)=\int_{0}^{V} p_{mn}(V')dV'$ (2)

The function$P_{mn}^{<}(V)$ can be determined experimentally from the histograms shown in figure 3,4 and 5 in extended data. This quantity can be obtained from the histograms by counting all the cells that show instabilities below the [m,n] mode. When we perform that treatment to the data a series of sigmoidal curves is obtained for each mode, which can be fitted quite well by an error function :

$P_{mn}^{<}(V)\to\frac{1}{2}ERFC\left(\frac{V-V_{mn}}{\sqrt{2}\sigma_{mn}}\right)$ (3)

With $ERFC(x)=\frac{2}{\sqrt{\pi}}\int_{X}^{\infty} e^{-t^{2}}dt$ (4)

This fit is consistent with transition probability functions having a Gaussian shape：

$p_{mn}(V)\cong\frac{1}{\sqrt{2\pi}\sigma_{mn}}\exp-\frac{1}{2}\left(\frac{V-V_{mn}}{\sigma_{mn}}\right)^{2}$ (5)

where the average transition voltage is $V_{mn}$ with a variance $\sigma_{mn}$.

## *Analysis of the images*

The video images are analyzed in terms of the sine basis functions that satisfy the boundary conditions but are not necessarily volume conserving. Now assume the image to be transformed into a set of grey-scales labeled for each point from [0,1]. The function of grey-scales will be denoted by $I(x,y)$ and is a function of lateral position. Define the Image Fourier coefficients $I_{mn}$ as follows:

$I_{mn}=\int_{0}^{L_{x}} dx\int_{0}^{L_{y}} dy I(x,y) Z_{mn}(x,y)$ (6)

The basic functions satisfying the boundary conditions $Z_{mn}(x,y)$are:

$Z_{mn}(x,y)=\sin\left(\frac{m\pi x}{L_{x}}\right)\sin\left(\frac{n\pi y}{L_{y}}\right)$ (7)

Based on the symmetry, in terms of number and positions of the holes in the oil film in the process of breaking, the dominant mode of rupture is decided.

## *Eigenvalue problem for finding volume conserving modes in Matlab (example for 150x315 μm^2^ cell)*

Matlab code to find the fundamental volume conserving modes by an eigenvalue problem. The mathematical basis underlying the code is found in the supplementary discussion:

Lx=150e-6; % width of cell in meter

Ly=315e-6; % length of cell in meter

V=input('Voltage='); % Input voltage

Nx=12; % number of fourier components in x direction

Ny=24; % number of fourier components in y direction

N=Nx.*Ny; % total number of fourier components

M=zeros(N+1,N+1); % Define a zero matrix (M in Supp. Disc.) of order n+1.

B=zeros(N+1,N+1); % Define a zero matrix (B in Supp. Disc.) of order n+1.

k2=2.2*8.85e-12.*V^2./22e-3./(5.5e-6+2.2/1.943*0.85e-6)^3;

% calculate value of K(V)^2 in 1/m2

for i=1:1:N % loop to fill first row of M (volume constraint)

m(i)=mod(i,Nx)+1; % designates 2D mode label m corresponding to i

n(i)=ceil(i/Nx); % designates 2D mode label n corresponding to i

M(1,i+1)=1/(m(i)*n(i)).*(1-(-1)^m(i)).*(1-(-1)^n(i))./4;

% Assign values to the first row of the matrix M.

% associated with volume conservation constraint

end

for j=1:1:N % loop to fill first column of M (pressure jump)

m(j)=mod(j,Nx)+1; % designates 2D mode label m corresponding to i

n(j)=ceil(j/Nx); % designates 2D mode label n corresponding to i

k1=pi^2.*(m(j)^2./Lx^2+n(j)^2./Ly^2);

% calculate K(mn)^2

M(j+1,1)=k1./k2.*(1-(-1)^m(j)).*(1-(-1)^n(j))./4;

% Assign values to the first column of the matrix M.

end

% loop to fill body of M and B except first column and row

% these are the growth rates of the pure sine modes

for i=1:1:N

for j=1:1:N

if i==j % only diagonal elements

m(i)=mod(i,Nx)+1; % designates 2D mode label m corresponding to i

n(i)=ceil(i/Nx); % designates 2D mode label n corresponding to i

k1=pi^2.*(m(i)^2./Lx^2+n(i)^2./Ly^2); % calculate K(mn)^2

M(i+1,j+1)=k1./k2-(k1./k2)^2;

% Assign value to the elements of the matrix M.

B(i+1,j+1)=1; % Assign value to the elements of the matrix B.

end

end

end

% After filling the matrices B and M,

% M(1,1) and B(1,1) = 0 as required

A=M^(-1)*B; % Define matrix for eigenvalue determination

[V,D] = eig(A); % Finding eigenvalues (D) and eigenvectors (V) of A

D=diag(D);

b=1;

for i=1:1:N % Extract positive eigenvalues and corresponding eigenvectors

if D(i,1)>=0

E(b)=D(i);

a(:,b)=V(:,i);

b=b+1;

end

end

## *Plotting of the theoretical unstable modes*

The initial time evolution of the oil film is expressed by fundamental volume conserving modes $\zeta_{mn}^{(conserved)}$ which are built up from sine modes that satisfy the pinning condition with amplitudes $\zeta_{ij}^{[mn]}$:

$\zeta_{mn}^{(conserved)}(x,y,t)=\sum_{i,j} \zeta_{ij}^{[mn]}(t)Z_{mn}(x,y)$ (8)

The oil film thickness is given by $h(x,y,t)=h+\zeta_{mn}^{(conserved)}(x,y,t)$. According to the Lambert-Beer law, the reflectivity distribution in the pixel can be described by$z=\frac{I}{I_{0}}=\exp(-\frac{2h(r)}{L_{abs}})$, where the absorption length *L_abs_* is 3.3 μm based on the extinction coefficient and dye concentration of the oil layer. The simulation mode images in this paper are made by applying a linear color map to the height function of the unstable mode in question. The minimum film thickness corresponds to white and the maximum dark blue. In this relatively simple way, sufficient resemblance with the experimental pictures are obtained.

## Supplementary discussion

## *Correcting for modes that are not strictly volume conserving*

Not all of the sine modes satisfying the pinning condition obey volume conservation, it therefore follows that these modes cannot exist in pure form. However certain superposition of the sine modes satisfying the pinning condition, which individually are not volume conserving can satisfy volume conservation. Consider a mode $\zeta_{mn}^{(conserved)}$ is built up from sine modes that satisfy the pinning condition with amplitudes $\zeta_{ij}^{[mn]}$:

$\zeta_{mn}^{(conserved)}(x,y,t)=\sum_{i,j} \zeta_{ij}^{[mn]}(t)\sin(k_{i}x)\sin(k_{j}y)$ (9)

In order to satisfy volume conservation the amplitudes must satisfy a certain condition. The enclosed volume of oil for such a mode is calculated as:

$$\frac{1}{L_{x}L_{y}}\int dxdy\zeta_{mn}^{conserved}(x,y,t)=\sum_{i,j=1} \frac{\zeta_{ij}^{[mn]}(t)}{\pi^{2}ij}(1-(-1)^{i})(1-(-1)^{j})$$

(10)

So volume conservation is ensured if

$\sum_{i,j=1} \frac{\zeta_{ij}^{[mn]}(t)}{\pi^{2}ij}(1-(-1)^{i})(1-(-1)^{j})=0$ (11)

A consistent method to deal with the volume conservation based on reference [20,21] in the manuscript can be used to determine the precise form of the unstable or fastest growing modes. This method is outlined as follows:

As explained in the main text, the total pressure in the region where the film is confined is written as:

$P(x,y,t)=\gamma\left[\nabla^{2}+k^{2}(V)\right]\varsigma_{mn}^{conserved}(x,y,t)+P_{0}$ (12)

In the rectangular region that confines the oil, a pressure jump acts to constrain the fluid into the cell. This pressure jump has a magnitude of $P_{0}$with respect to the reference pressure and its value will be determined to be consistent with volume conservation. Formulated in Fourier components we can write the pressure jump as:

$P_{0}=P_{0}\sum_{m,n=1} b_{mn}\sin k_{m}x\sin k_{n}y$ (13)

The Fourier coefficients are given by:

$b_{mn}=\frac{(1-(-1)^{m})(1-(-1)^{n})}{\pi^{2}mn}$ (14)

Now the dynamic equation for the initial perturbation is given by:

$\frac{\partial\zeta(x,y,t)}{\partial t}=-\frac{h_{0}^{3}}{3\eta}\nabla^{2}P(x,y,t)$ (15)

where the pressure on the right hand side is given by equation (12). To find the precise form of the volume conserving modes and their respective growth rates, expand the interface perturbation as follows:

$\zeta(x,y,t)=\sum_{i,j} \zeta_{ij}(t)\sin(k_{i}x)\sin(k_{j}y)$ (16)

Then inserting in (15) while making use of (12), (13) and (14) leads to:

$\frac{\partial\zeta_{mn}(t)}{\partial t}=\frac{h^{3}\gamma}{3\eta}\left\{(k_{mn}^{2}k^{2}(V)-k_{mn}^{4})\zeta_{mn}(t)-b_{mn}k_{mn}^{2}\frac{P_{0}(t)}{\gamma}\right\}$ (17)

For the dynamical system the pressure jump can be determined through the volume conservation constraint:

$\sum_{i,j=1} \frac{\zeta_{mn}(t)}{\pi^{2}mn}(1-(-1)^{m})(1-(-1)^{n})=0$ (18)

Now equations (17) and (18) form a closed linear equation system for the vector:

$\mathbf{Z}(T)=\left(\frac{P_{0}(t)}{\gamma k^{2}(V)},\zeta_{11}(t),\zeta_{12}(t),...\zeta_{mn}(t),....\right)$ (19)

Here we use a dimensionless timescale defined as:

$T=\frac{h^{3}\gamma}{3\eta k^{4}(V)}t$ (20)

Note that we are switching indices such that the index of the linear system labels corresponds to the 2 dimensional sine modes pinned at the pixel wall height.

$\mathbf{B}\cdot\frac{\partial\mathbf{Z}(T)}{\partial T}=\mathbf{M}\cdot\mathbf{Z}(T)$ (21)

Now starting the index with zero the matrices B and M are given by:

$\begin{matrix} B_{00}=0 \\ B_{ij}=\delta_{ij}\text{ for }i\neq0\text{ or j}\neq0 \end{matrix}$ (22)

$\begin{matrix} M_{00}=0 \\ M_{i0}=b_{mn}Q_{mn}\text{ for }i\neq0\text{ or j=}0 \\ M_{0j}=b_{mn}\text{ for }i=0\text{ or j}\neq0 \\ M_{ij}=\delta_{ij}\left(Q_{mn}-Q_{mn}^{2}\right)\text{ for }i\neq0\text{ or j}\neq0 \end{matrix}$ (23)

This system of linear first order differential equations can be solved by matrix manipulation searching for modes with a growth rate $s$: $\mathbf{Z}(T)=\mathbf{Z}(0)e^{sT}$

$\mathbf{M}^{-1}\cdot\mathbf{B}\cdot\mathbf{Z}(T)=s^{-1}\mathbf{Z}(T)$ (24)

Finding the eigenvectors and eigenvalues of $\mathbf{M}^{-1}\cdot\mathbf{B}$gives us the unstable modes (in case of a positive eigenvalue) and the corresponding growth times $s^{-1}$ respectively. With the aid of the matlab code presented in supplementary methods 3, we solve Eq. (24) numerically. By finding the points of zero growth of the volume conserving modes, we can find the marginally stable transition points. In Supplemental Figure 6 one finds a graphical depiction of the unstable mode for the square pixel, using the theoretical analysis described above.

The results are tabularized in that table 1. It can be seen that apart from the [1,3] mode of the square cell, the corrections due to volume conservation constraint are small. In table 1 we have also determined the points where the modes take over as fastest growing (closed circles in the manuscript Figure 4). In some cases the crossover between the rates was difficult to obtain accurately. In those instances we took values from the analysis that was not volume conserving.

Table 1: Marginal stability transitions with and without correction for volume conservation. The analysis tabularized for the experimentally observed modes only.

Table 2: Determination of dynamic transition point [m,n]->[m’,n’], defined as the point where the [m’,n’] mode becomes faster compared to the [m,n] mode. [-] indicates difficulties determining the transition sufficiently accurately with the numerical method.

## Supplementary video

## *1) Modes transition with applied voltage for square pixels (150×150 μm^2^)*

The view area under the high-speed camera was of 5×9 pixels. With the increase in applied voltage, the oil film develops from flat to modes [1,2], [1,3] and [3,3] in sequence. According to the data analysis methods described in “Fit procedure for data” of supplementary information, the transition voltages for the modes [1,2], [1,3] and [3,3] are approximately 32V, 39V and 55V, respectively.

## *2) Modes transition with applied voltage for rectangular pixels (150×315 μm^2^)*

The view area under the high-speed camera was of 7×7 pixels. With the increase in applied voltage, the oil film develops from flat to modes [1,2], [1,3], [1,4], [1,5], [1,7] and [2,5] in sequence, which is consistent with our theoretical predictions. According to the data analysis methods described in “Fit procedure for data” of supplementary information, the transition voltages for the modes [1,2], [1,3], [1,4], [1,5], [1,7] and [2,5] are approximately 16V, 17V, 24V, 28V, 36V and 45V, respectively.

## *3) Modes transition with applied voltage for rectangular pixels (67.5×315 μm^2^)*

The view area under the high-speed camera was of 5×9 pixels. With the increasing in applied voltage, the oil film develops from flat to modes [1,2], [1,3],[1,5] and [1,7] in sequence. According to the data analysis methods described in “Fit procedure for data” of supplementary information, the transition voltages for the modes [1,2], [1,3],[1,5] and [1,7] are approximately 30V, 35V, 42V and 55V, respectively.

## Supplementary figures (Extended Data)


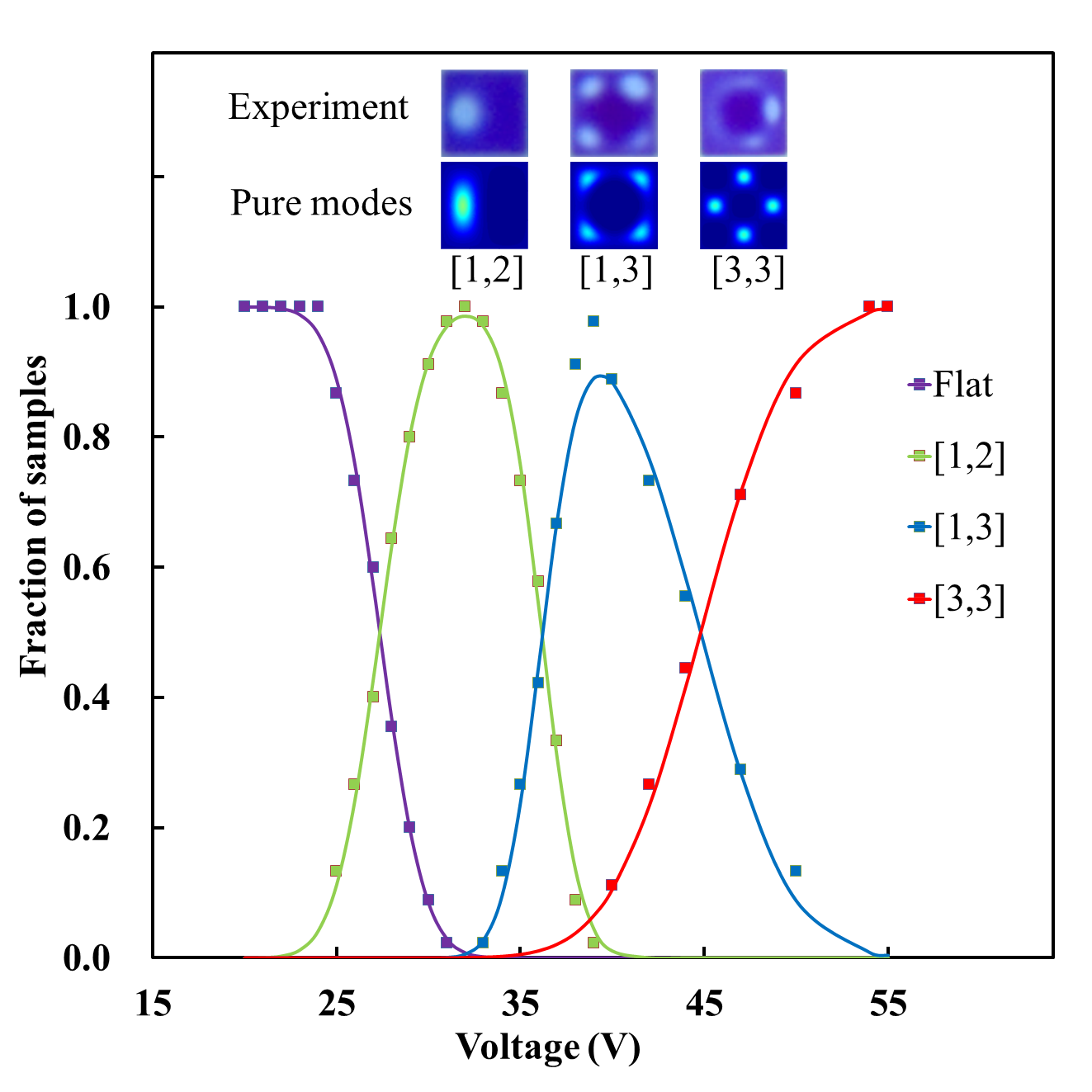


**Figure 1 ︱The observed count for each mode as a function of applied voltage of the 150x150 µm^2^ cell.** Symbols represent processed experimental data and lines to corresponding data fits as described in the Supplementary Information section 2.1. Inserts at top show typical experimental images and corresponding pure modes generated by model calculation (Supplementary Information section 2.3).


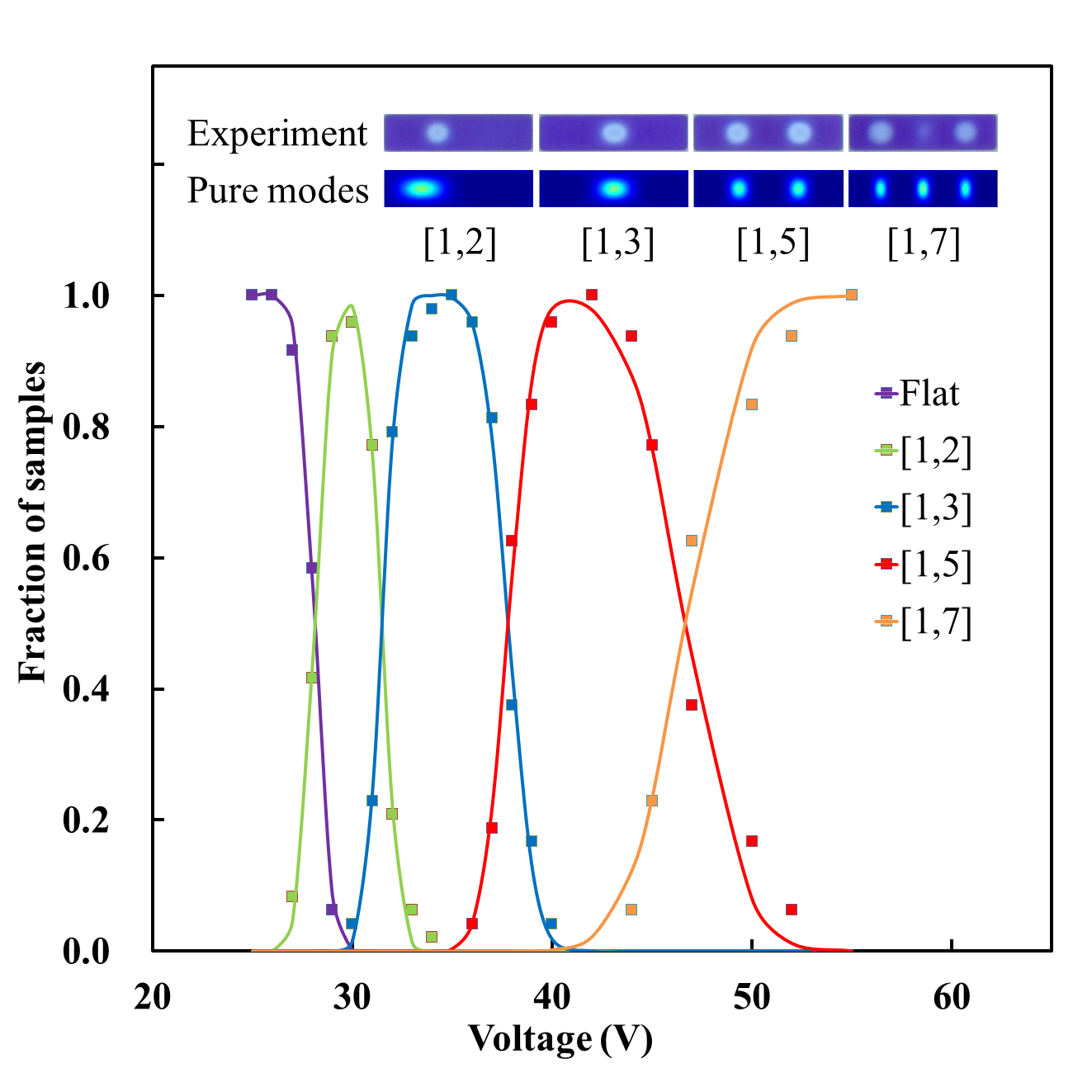


**Figure 2 ︱The observed count for each mode as a function of applied voltage of the 67.5x315 μm^2^ cell.** Symbols represent processed experimental data and lines to corresponding data fits as described in the Supplementary Information section 2.1. Inserts at top show typical experimental images and corresponding pure modes generated by model calculation (Supplementary Information section 2.3).

**
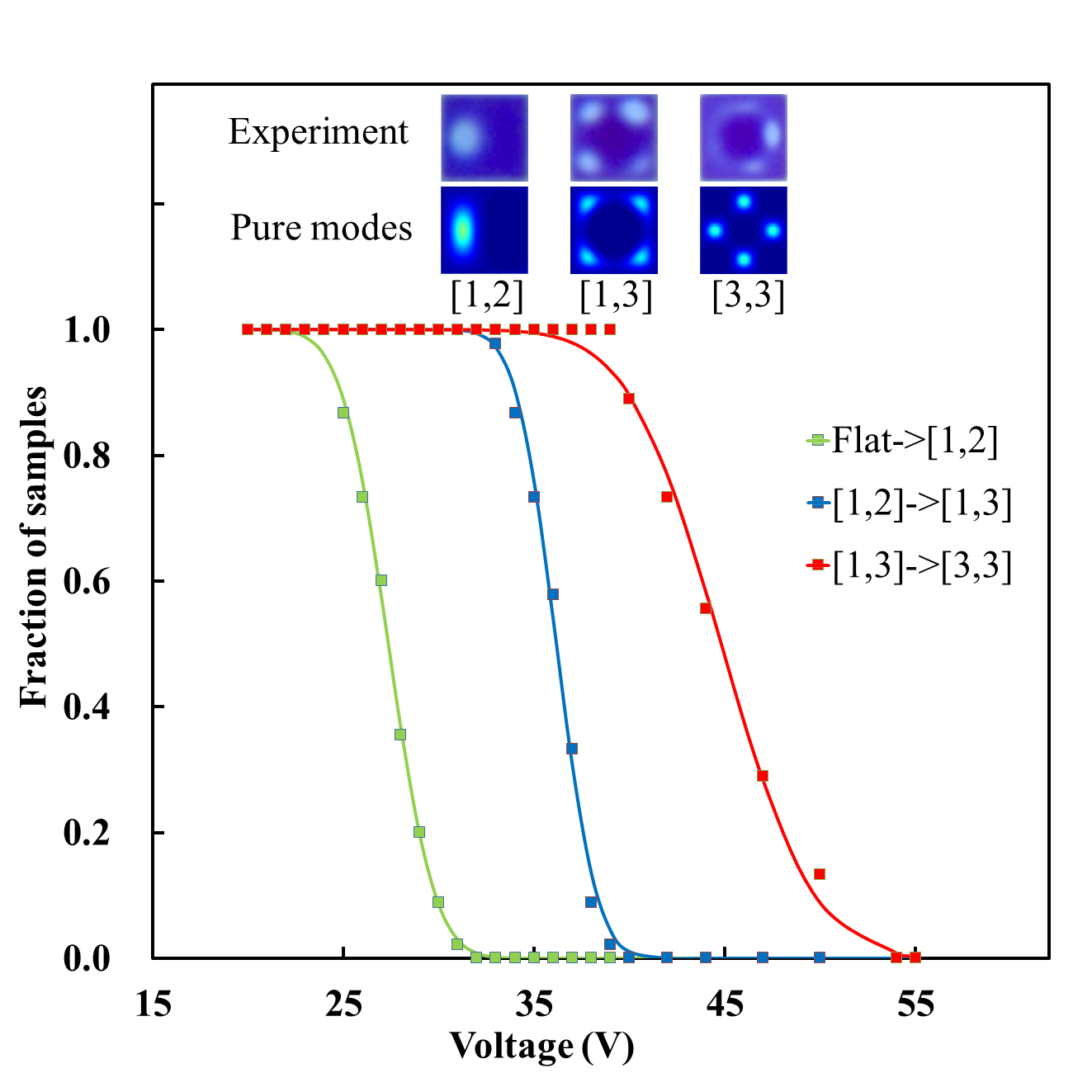
**

**Figure 3 ︱Modes statistics with the increasing applied voltage of the sample with 150x150 μm^2^ cells.** Symbols represent processed experimental data and lines to corresponding data fits as described in the Supplementary Information section 2.1. Inserts at top show typical experimental images and corresponding pure modes generated by model calculation (Supplementary Information section 2.3).


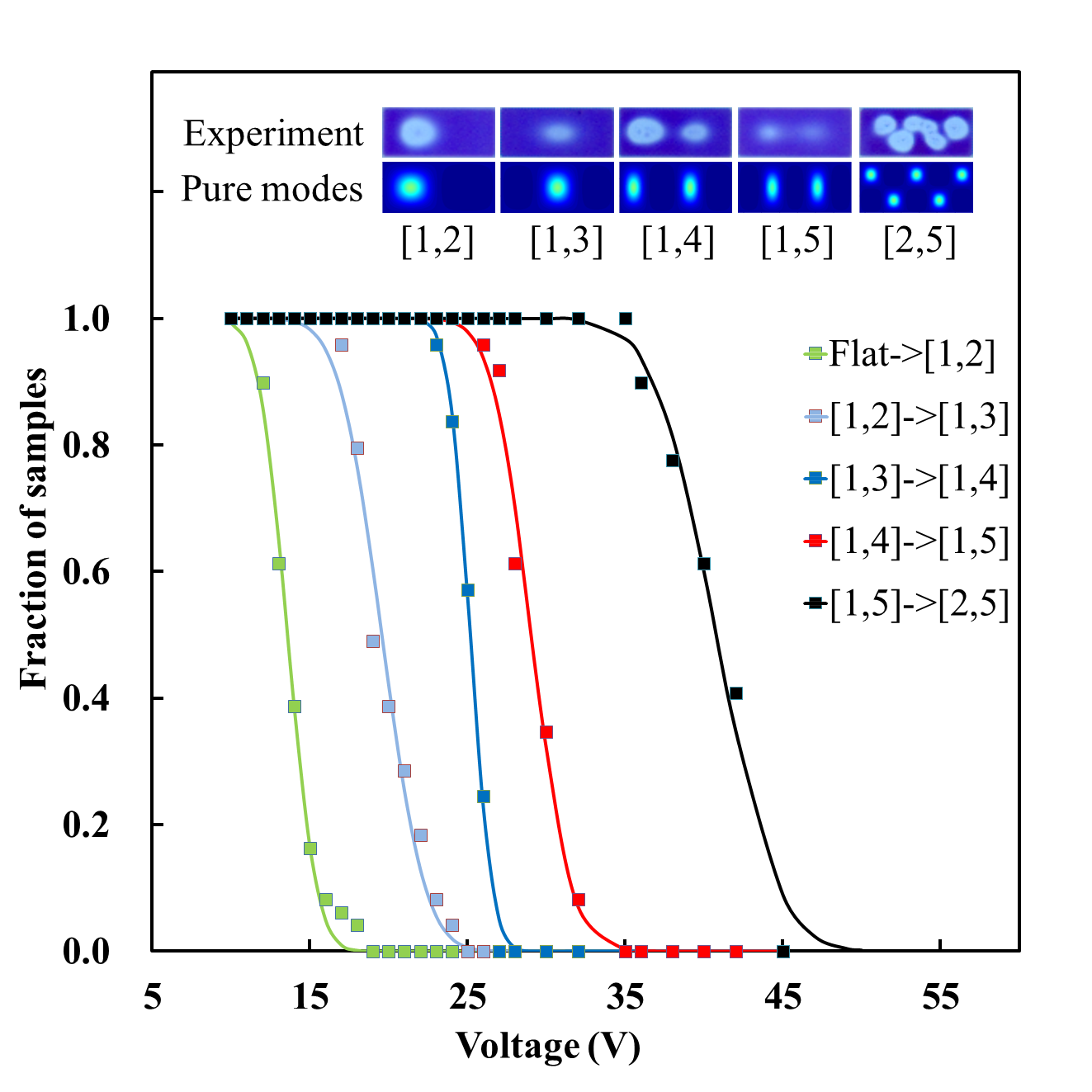


**Figure 4 ︱Modes statistics with the increasing applied voltage of the sample with 150x315 μm^2^ cells.** Symbols represent processed experimental data and lines to corresponding data fits as described in the Supplementary Information section 2.1. Inserts at top show typical experimental images and corresponding pure modes generated by model calculation (Supplementary Information section 2.3).


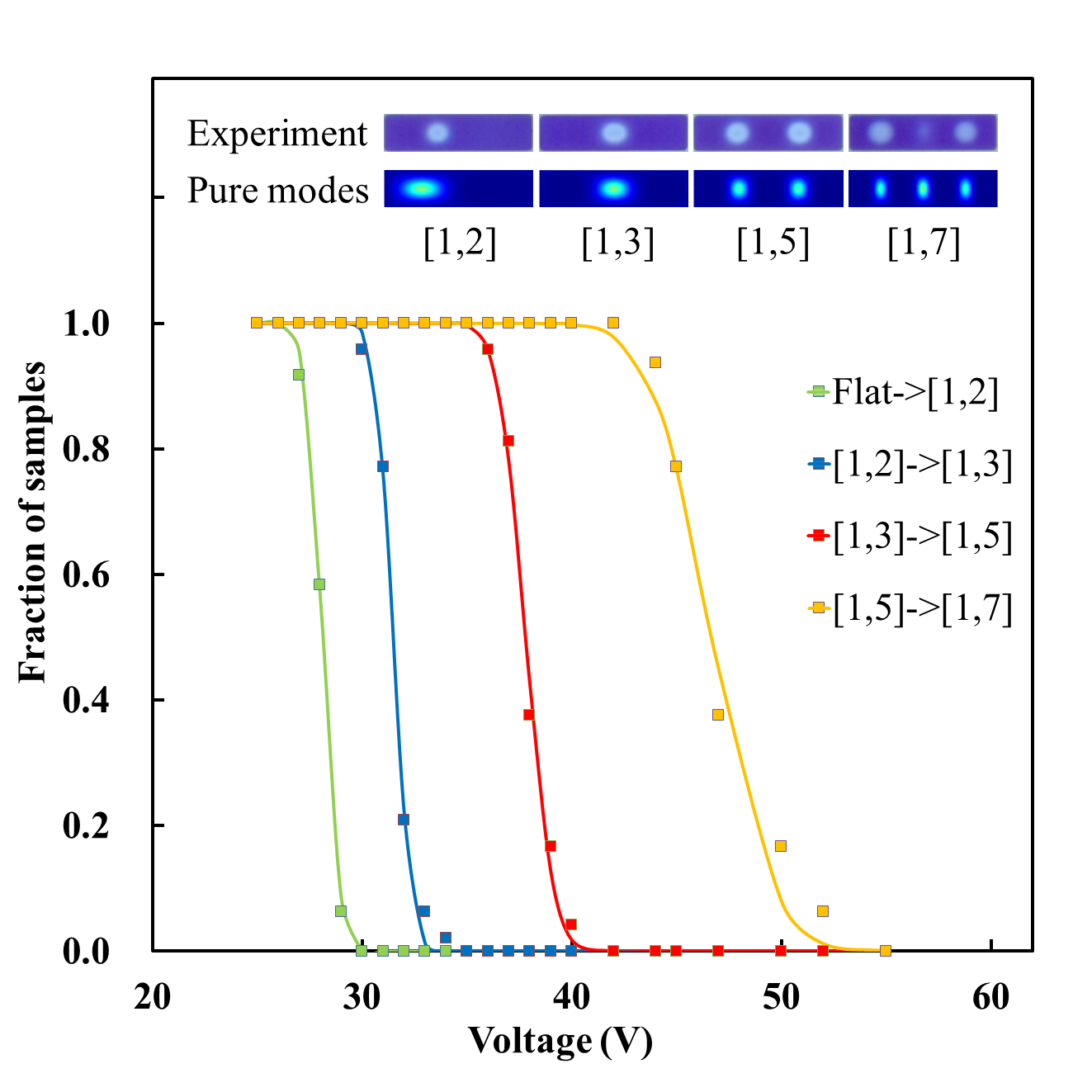


**Figure 5 ︱Modes statistics with the increasing applied voltage of the sample with 67.5x315 μm^2^ cells.** Symbols represent processed experimental data and lines to corresponding data fits as described in the Supplementary Information section 2.1. Inserts at top show typical experimental images and corresponding pure modes generated by model calculation (Supplementary Information section 2.3).

**Figure 6 ︱Depiction of the fundamental volume conserving unstable modes of on a 150x150 μm^2^ cell.** From left to right the [1,2], [1,3] (rather [1,3]+[3,1]) and the [3,3] mode are shown. On the x,y axis the lateral position of the cell is indicated, while the vertical (z-axis) indicates the height of the oil film. Note that the [1,3] mode has depletion of oil all around the boundary.
